# Effectiveness of exercise modalities on breast cancer patient outcomes: a systematic review and meta-analysis

**DOI:** 10.1186/s40959-024-00235-z

**Published:** 2024-06-18

**Authors:** Naser Yamani, Aymen Ahmed, Mohammad Khan, Zachary Wilson, Muteia Shakoor, Syeda Fizza Qadri, Samuel Unzek, Marc Silver, Farouk Mookadam

**Affiliations:** 1 Division of Cardiology, Banner University Medical Center, University of Arizona, Phoenix, AZ USA; 2 Department of Medicine, Dow University of Health Sciences, Karachi, Pakistan

**Keywords:** Breast neoplasm, Physical activity, Health-related quality of life, Muscle strength, Fatigue, Physical function

## Abstract

**Background:**

The effects of exercise in patients with breast cancer (BC), has shown some profit, but consistency and magnitude of benefit remains unclear. We aimed to conduct a meta-analysis to assess the benefits of varying types of exercises in patients with BC.

**Methods:**

Literature search was conducted across five electronic databases (MEDLINE, Web of Science, Scopus, Google Scholar and Cochrane) from 1st January 2000 through 19th January 2024. Randomized controlled trials (RCTs) assessing the impact of different types of exercise on outcomes related to fitness and quality of life (QOL) in patients with BC were considered for inclusion. Outcomes of interest included cardiorespiratory fitness (CRF), health-related quality of life (HRQOL), muscle strength, fatigue and physical function. Evaluations were reported as mean differences (MDs) with 95% confidence intervals (CIs) and pooled using random effects model. A p value < 0.05 was considered significant.

**Results:**

Thirty-one relevant articles were included in the final analysis. Exercise intervention did not significantly improved the CRF in patients with BC when compared with control according to treadmill ergometer scale (MD: 4.96; 95%Cl [-2.79, 12.70]; *P* = 0.21), however exercise significantly improved CRF according to cycle ergometer scales (MD 2.07; 95% Cl [1.03, 3.11]; *P* = 0.0001). Physical function was significantly improved as well in exercise group reported by 6-MWT scale (MD 80.72; 95% Cl [55.67, 105.77]; *P* < 0.00001). However, exercise did not significantly improve muscle strength assessed using the hand grip dynamometer (MD 0.55; 95% CI [-1.61, 2.71]; *P* = 0.62), and fatigue assessed using the MFI-20 (MD -0.09; 95% CI [-5.92, 5.74]; *P* = 0.98) and Revised Piper scales (MD -0.26; 95% CI [-1.06, 0.55] *P* = 0.53). Interestingly, exercise was found to improve HRQOL when assessed using the FACT-B scale (MD 8.57; 95% CI [4.53, 12.61]; *P* < 0.0001) but no significant improvements were noted with the EORTIC QLQ-C30 scale (MD 1.98; 95% CI [-1.43, 5.40]; *P* = 0.25).

**Conclusion:**

Overall exercise significantly improves the HRQOL, CRF and physical function in patients with BC. HRQOL was improved with all exercise types but the effects on CRF vary with cycle versus treadmill ergometer. Exercise failed to improve fatigue-related symptoms and muscle strength. Large RCTs are required to evaluate the effects of exercise in patients with BC in more detail.

**Supplementary Information:**

The online version contains supplementary material available at 10.1186/s40959-024-00235-z.

## Introduction

Breast cancer (BC) is one of the most prevalent cancers globally. Approximately 2.3 million people were diagnosed with BC in 2020, and this number is expected to rise by 40% in 2040, with the younger demographic being increasingly affected [1]. Around 685,000 patients with BC died in 2020, and the mortality burden associated with BC remains concerning due to delayed diagnoses, treatment and later stages of the disease at presentation [1]. Numerous strategies have been developed for the prevention and treatment of BC, with regular exercise being one of the most crucial components. According to the American College of Sports Medicine (ACSM) guidelines, performing at least 30 min of daily physical activity contributes to decreased likelihood of developing BC [2]. As for the patients with BC, ACSM has recommended 150 min of aerobic activity and resistance training for two or more days per week [3].However, these patients suffer from detrimental health effects and excessive fatigue during and following cancer treatment which greatly impairs their physical functioning, reduces cardiorespiratory fitness (CRF) and muscle strength, ultimately resulting in a poor health-related quality of life (HRQOL) [4, 5]. In order to alleviate these symptoms, patients tend to rest and reduce their level of physical activity.

In 2017, Dickinson et al. [6] found that indulging in daily exercise aids in counteracting the muscular degeneration and dysfunction induced by doxorubicin (DOX) which is among the most effective drugs for treating BC. Numerous other trials and articles have also indicated that exercise is associated with improved symptoms of cancer treatment, leading to a better quality of life (QOL). However, current literature harbors conflicting results with respect to exercise intervention in patients with BC during and after treatment [7]. Although a meta-analysis by McNeelyet al. [8] in 2006 reported that exercise improved QOL, peak oxygen consumption and physical functioning, and reduced fatigue symptoms in patients with BC. Since that time however a large number of trials that have been published, that may temper the earlier results from McNeely et al. Recent meta-analyses conducted on similar topics suffer from poor quality of data assessment and outcome processing. For instance, a meta-analysis published by Furmaniak et al. in 2016 [9], emphasized that exercise is a crucial factor in eradicating the adverse effects of cancer treatment, but they reported data by merging different outcomes together and assessed it with different scales which may have led to some bias in their results.

Given the potential variations in prior studies and the absence of a recent comprehensive analysis summarizing trial-based evidence, we aimed to assess the impacts of various exercise modalities (aerobic, resistance or both combined) on QOL and health-based parameters in patients with BC during and after treatment.

## Materials and methods

This systematic review and meta-analysis has been reported in accordance with guidelines provided by preferred reporting items for systematic review and meta-analyses (PRISMA) [10]. The protocol for this meta-analysis has been registered on PROSPERO and can be accessed at www.crd.york.ac.uk/PROSPERO/display_record.asp?ID=CRD42024525640. Approval from the institutional review board was not required since the data was publicly available.

### Data sources and search strategy

A comprehensive search was conducted across MEDLINE, Web of Science, Scopus, Google Scholar and Cochrane databases to search for randomized controlled trials (RCTs) assessing the effects of different types of exercises on post-treatment cancer outcomes in patients with BC from 1st January 2000 to 19th January 2024, without any language restrictions. The search was carried out by using the words “breast cancer”, or any synonym including “breast cancer survivors”. In addition, exercise was searched using the words “physical activity”, “physical training.” To enhance the search, Boolean operators (AND, OR) were applied. The detailed search strategy for each database is provided in Supplementary Table 1.

### Study selection and data extraction

All articles retrieved from the systematic search were exported to EndNote Reference Manager (Version X7.5; Clarivate Analytics, Philadelphia, Pennsylvania) where duplicates were sought and removed. The remaining articles were assessed at title and abstract level by two independent investigators (SFQ and MS), after which full text were read to confirm relevance. Any disagreements were resolved by discussion with a third investigator (AA).

The following pre-defined inclusion criteria was used: (a) RCTs which investigated the effects of exercise on health-related outcomes in patients with BC during treatment and post-treatment, regardless of their gender and grade of cancer, (b) Exercises involving aerobic, resistance, or a combination of both involving body stretching, use of treadmill or cycle ergometer, except any aquatic exercises. (c)compared the exercise group of patients with those who had undergone usual/standard care (control group), (d) had a minimum follow-up of one month.

### Data extraction and outcomes of interest

Data extraction was done by the authors SFQ and MS on a pre-specified data extraction sheet. Data extracted from the studies included the publication year, sample size, mean age of the intervention and the control group, the intervention provided, duration, intensity and frequency of the intervention, number of patients in the control and the intervention group and the scales used for assessing the outcomes. Outcomes of interest were patient-reported improved CRF, fatigue, physical function, muscle strength and hence the overall improvement in the QOL. Each outcome was measured with certain scales, HRQOL (Functional Assessment of Cancer Therapy-Breast; FACT-B and European Organization for the Research and Treatment of Cancer Quality of Life; EORTIC QLQ-C30), Fatigue (Multidimensional Fatigue Inventory; MFI-20 and Revised Piper Fatigue scale), Physical function (6 min Walking Test; 6-MWT), CRF (peak oxygen uptake in ml/kg/min by treadmill and cycle ergometer) and Muscle strength by hand grip dynamometer.

### Statistical analysis

The software used for analysis is Review Manager (Version 5.4.1) [11]. Continuous variables were reported as mean differences (MDs) with corresponding 95% confidence intervals (CIs), and were pooled using a generic inverse variance random-effects model. The I^2^ statistic was used to evaluate heterogeneity across studies, and a value of I^2^ = 25-50% was considered mild, 50-75% as moderate and I^2^ > 75% as severe [12]. A p-value < 0.05 was considered statistically significant in all cases.

### Risk of bias assessment

**Fig. 1:**
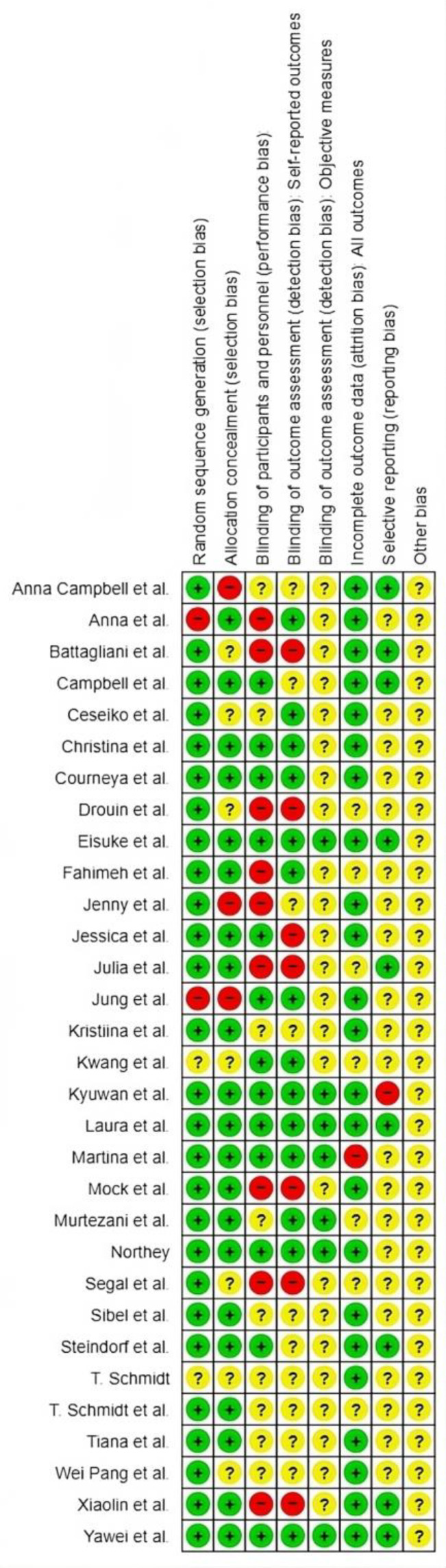
Risk of bias assessed by Rob

The methodological risk of bias assessment of all RCTs was evaluated according to Revised Cochrane Risk of Bias tool (Rob2) [13]. Studies were assessed based on the following domains: selection bias, performance bias, detection bias, attrition bias, reporting bias and other bias, and were categorized in each domain on the basis of low risk (+), unclear risk (?) and high risk (-). Figure 1 shows detailed assessment of risk of bias.

## Results

### Literature search

**Fig. 2:**
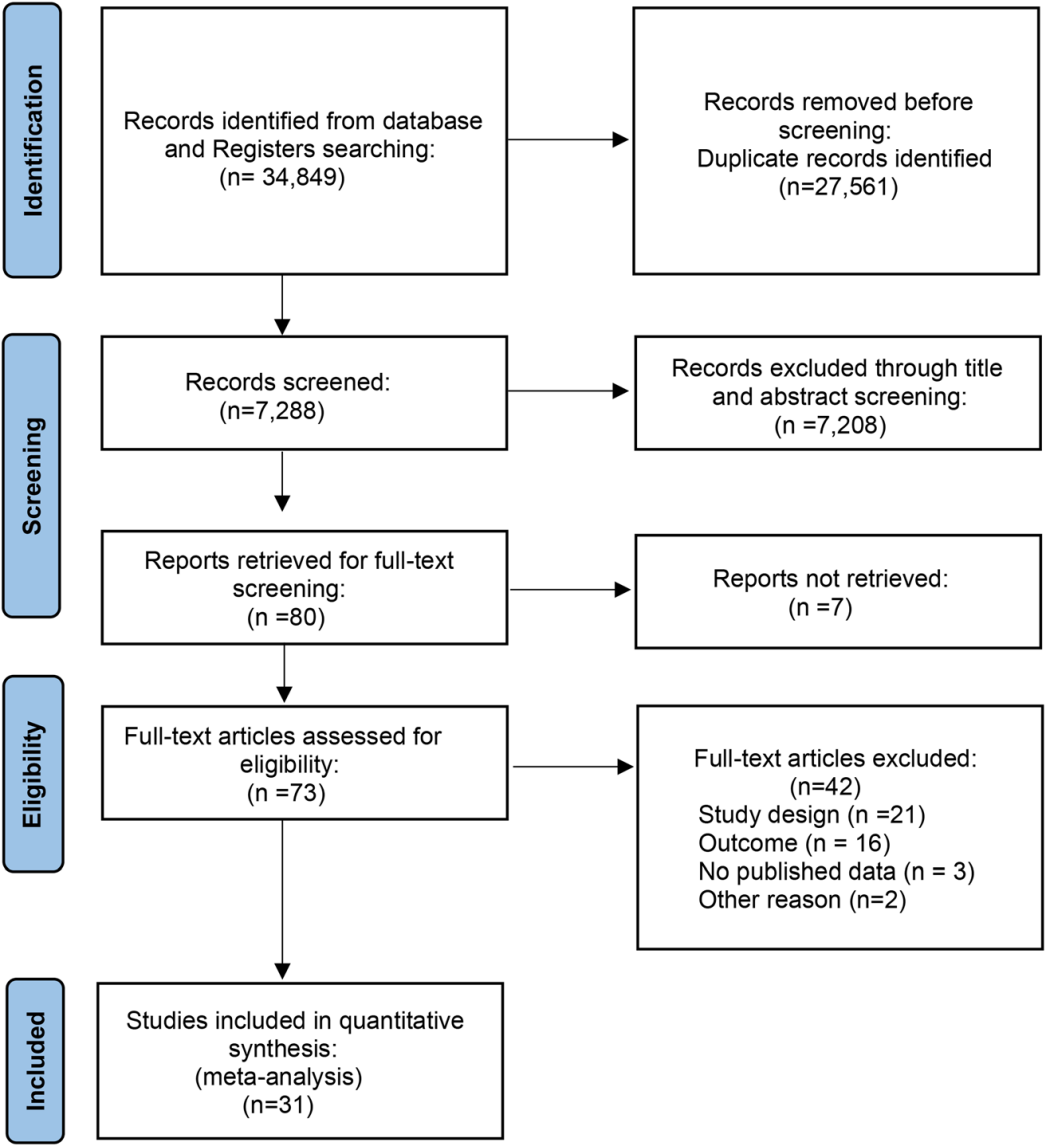
The PRISMA flow chart summarizing literature search results

The initial searching of database and registers yielded 34,849 studies. After title, abstract and duplicates screening, 73 articles were identified for full-text assessment. Further, 42 full-text articles were excluded based on issues with study design, irrelevant outcomes, no published data, and other reasons including non-availability of article in English language. Thus, 31 articles were found potentially relevant for the final analysis. The summary of our literature search results is presented in the PRISMA flow chart (Fig. 2).

### Study characteristics and risk of bias assessment

A total of 3,059 patients with BC were involved in analysis, out of which majority (85.7%) were female participants. The mean age of participants involved in exercise (intervention) and control group is 52.6 (standard deviation [SD]: 9.0) and 52.7 (SD: 8.25) respectively. The mean BMI at baseline of exercise (intervention group) and control group is 25.24 (SD: 4.43) and 25.41 (SD: 3.98) respectively. The summary of study characteristics and participants demographics are presented in Table 1. The risk of bias across the included studies was unclear (Fig. 1). Visual inspection of funnel plots suggested at least 10 studies with publication bias (Supplementary Fig. 1A, B).

**Table 1:** Summary of study and patient characteristics

Study	Study design	Sample size	Participants		Mean age (mean, Sd)		Mean BMI		Patients lost to follow up		Intervention	Duration of intervention /weeks	Intensity of intervention	Outcome
			Intervention	Control	Intervention	Control	Intervention	Control	Intervention	Control				
Segal et al., [14]	Randomized controlled trial	123	82	41	51.2 (8.7)	50.3 (8.7)			0	0	Supervised aerobic exercise	26 weeks	50–60% of the maximal oxygen uptake	HRQOL, assessed using FACT B scale
Drouin et al., [15]	Randomized controlled trial	21	13	10	49.4(7.0)	51.9(10.0	-	-	-	-	Aerobic exercise	7 weeks	50–70% of maximum heart rate	Fatigue assessed by Revised Piper and CRF by treadmill ergometer
Courneya et al., [16]	Prospective, randomized controlled trial	52	24	28	59 (5)	58 (6)	29.4 (7.4)	29.1 (6.1)	1	0	Aerobic exercise, cycle ergometers	15 weeks	70–75% of maximal oxygen consumption.	HRQOL, assessed using FACT B scale
Battagliani et al., [17]	Randomized controlled trial	20	10	10	-	-	-	-	-	-	Mixed aerobic and resistance	15 weeks	40–60% predicted exercise capacity	Fatigue assessed by Revised Piper scale
Mock et al., [18]	Randomized controlled trial	119	60	59	51.3(8.9)	51.6 (9.7)	25.5(4.0)	25.8(5.1)	6	2	Aerobic exercise	6 weeks	50–70% of maximum heart rate	Fatigue assessed by Revised Piper scale
Anna Campbell et al., [19]	Randomized controlled trial	19	12	10	48 (10)	47 (5)	-	-	-	-	Mixed aerobic and resistance exercise	12 weeks	60–75% of maximum heart rate	HRQOL, assessed using FACT B scale
Tinna et al., [20]	Prospective, non- blinded randomized two-arm phase III trial	500	263	237	52.3 (8)	52.4 (8.25)			21	16	Aerobic exercise	52 weeks	86–92% of maximal heart rate	HRQOL, assessed using EORTC QLQ- C30 scale
T. Schmidt et al., [21]	Prospective, randomized study	38	15	18	58 (8.4)	55 (10.59)			-	-	Aerobic exercise	26 weeks	-	HRQOL, assessed using EORTC QLQ- C30 scale
Laura et al., [22]	Randomized controlled trial	222	105	108	54.9 ( 9.3)	53.9 (7.7)			0	0	Exercise, non specified	13 weeks	40–59% of of maximum heart rate to 40–59% of of maximum heart rate	HRQOL, assessed using FACT B scale
Martina et al., [23]	Prospective, randomized, controlled trial	95	49	46	52.2 (9.9)	53.3 (10.2)	25.7 (4.6)	26.3 (4.9)	3	3	Resistance exercise	12 weeks	-	HRQOL, assessed using EORTC QLQ- C30 scale
Steindorf et al., [24]	Prospective, randomized, controlled trial	160	80	80	55.2 (9.5)	56.4 (8.7)	26.9 (5.4)	27.6 (4.8)	3	2	Resistance exercise	12 weeks	-	HRQOL, assessed using EORTC QLQ- C30 scale
Murtezani et al., [25]	Prospective, randomized controlled trial	62	30	32	53 (11)	51 (11)	25.9 (2.8)	26.0 (3.3)	7	4	Aerobic exercise	10 weeks	50–75% hear rate reserve	HRQOL, assessed using FACT B scale
Thorsten et al., [26]	Prospective, controlled and randomized intervention trial	81			56(10.15)	54(11.9)			12	2	Combined exercise.	12 weeks	-	HRQOL, assessed using EORTC QLQ- C30 scale. Fatigue assesed using MFI-20 scale.
Campbell et al., [27]	Randomized controlled trial	19	10	9	53.2(7.0)	51.4(5.1)	26.3(5.7)	26.1(5.5)	3	2	Aerobic exercise	24 weeks	From 60–80% heart rate reserve	CRF assesed using treadmil
Christina et al., [28]	Randomized controlled trial	100							4	5	Combined exercise	17 weeks	50 and 70% heart rate maximum	HRQOL, assessed using FACT B scale. CRF assessed using treadmil.
Cešeiko et al., [29]	Randomized controlled trial	55	27	28	48.2 (6.7)	49.0 (8.0)			-	-	Resistance exercise	12 weeks	-	HRQOL, assessed using EORTC QLQ- C30 scale
Northey et al., [30]	3 arm, randomized controlled trial	17	6	6	60.3 (8.1)	61.5 (7.8)			0	0	Aerobic exercise	12 weeks	90% of maximum heart rate	CRF assesed using cycle ergometer
Jessica et al., [31]	Parallel-group randomized trial	174	58	57	59(9)	58(9)	30.1(6)	29.1(5.2)	11	7	Aerobic exercise	16 weeks	alternated among intensities of 55%, 65%, 75%, 80%, and > 95% of maximal oxygen consumption	HRQOL, assessed using FACT B scale. CRF assessed using treadmil.
Julia et al., [32]	Randomized controlled trial	63	31	32	51.32 ( 10.15)	48.33 ( 7.72)			5	4	Aerobic exercise	24 weeks	-	HRQOL, assessed using EORTC QLQ- C30 scale
Kyuwan et al., [33]	Single-center, pilot randomized controlled trial	30	15	15					0	0	Aerobic exercise	8 weeks	Individually prescribed based on maximal oxygen consumption	HRQOL, assessed using FACT B scale. Fatigue assessed using MFI20 scale. Physical function assessed using 6MTW scale.
Sibel et al., [34]	prospective, single-blind, randomized trial	31	15	16	51.40 (10.6)	50.7 (7.6)	26.0 (4.9)	24.5(3.4)	0	0	Yoga	10 weeks	-	HRQOL, assessed using EORTC QLQ- C30 scale
Jenny et al., [35]	randomized, controlled, prospective interventional PhysSURG-B trial	287	139	148					57	46	Aerobic exercise	6 weeks	-	HRQOL, assessed using FACT B scale
Yawei et al., [36]	single-blind randomized parallel controlled tria	200	Aerobic = 49, Resistance = 47	48	aerobic = 47.37 (9.99), resistance = 49.38 (9.51)	51.69 (10.14)	aerobic = 22.92 (3.27), resistance = 23.90 (4.37)	23.90 (3.85)	6	2	Combined exercises	26 weeks	60–80% of the maximum heart rate	HRQOL, assessed using FACT B scale
Eisuke et al., [37]	parallel-group, single-blind, randomised controlled trial	50	21	23	48 (6)	49 (5)	21.0 (2.2)	20.9 (2.0)	7	6	Resistance exercise	12 weeks	-	CRF assesed using cycle ergometer
Xialon et al., [38]	single-blinded randomized controlled trial	70	35	35			22.86 (2.55)	23.26 (2.56)	3	0	Baduanjin exercise	12 weeks	-	HRQOL, assessed using FACT B scale
Kwang et al., [39]	Randomized controlled trial	30	15	15	54.7 ( 5.1)	55.4 ( 4.3)	23.3 ( 3.26)	24.4 ( 3.34)	2	3	Resistance exercise	12 weeks	-	Muscle strength assessed using hand grip dyanometer
Wei-Pang et al., [40]	Randomized controlled trial	32	16	13	52.4 (8.9)	50.3 (7.7)	24.6 (6.1)	23.2 (2.7)	0	3	Combined exercise	13 weeks	70–75% of maximal oxygen consumption.	CRF assesed using cycle ergometer
Fahimeh et al., [41]	double-blinded randomized controlled trial,	76	35	35	38.14 (10.70)	42.63 (8.11)					Aerobic exercise	5 weeks		HRQOL, assessed using EORTC QLQ C30 scale. Physical function assessed using 6MTW scale.
Anna et al., [42]	non-blinded randomized controlled trial	577	288	89	58 (12)	58 (11)			15	6	Combined exercise	12 weeks	40–50% of the heart rate reserve	HRQOL, assessed using EORTC QLQ- C30 scale
Kristiina et al., [43]	Randomized controlled trial	446	106	446	52(7)	53(8)	24.6(3.5)	25.9 (4.1)	-	-	Aerobic exercise	52 weeks	-	HRQOL, assessed using EORTC QLQ-C30 scale.
Jung et al., [44]	prospective, randomized, controlled study	60	28	29	46.9 ( 8.3)	48.1 ( 7.9)			2	1	Combined exercise	4 weeks	40% of maximal oxygen consumption	HRQOL, assessed using EORTC QLQ-C30 scale.

### Meta-analysis results

**Fig. 3:**
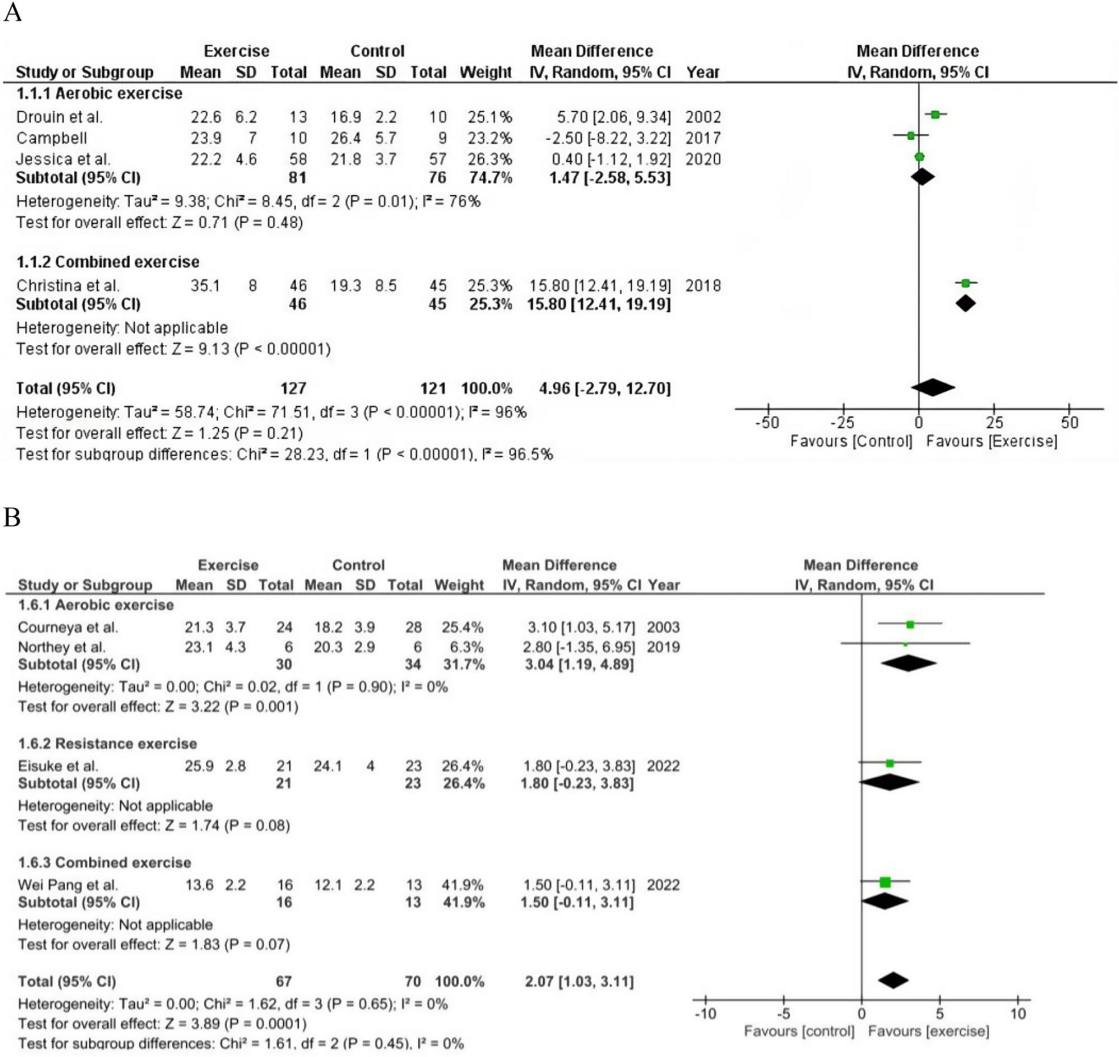
**A** pooled results of CRF by treadmill ergometer. **B** Pooled results of CRF by cycle ergometer

**Fig. 4:**
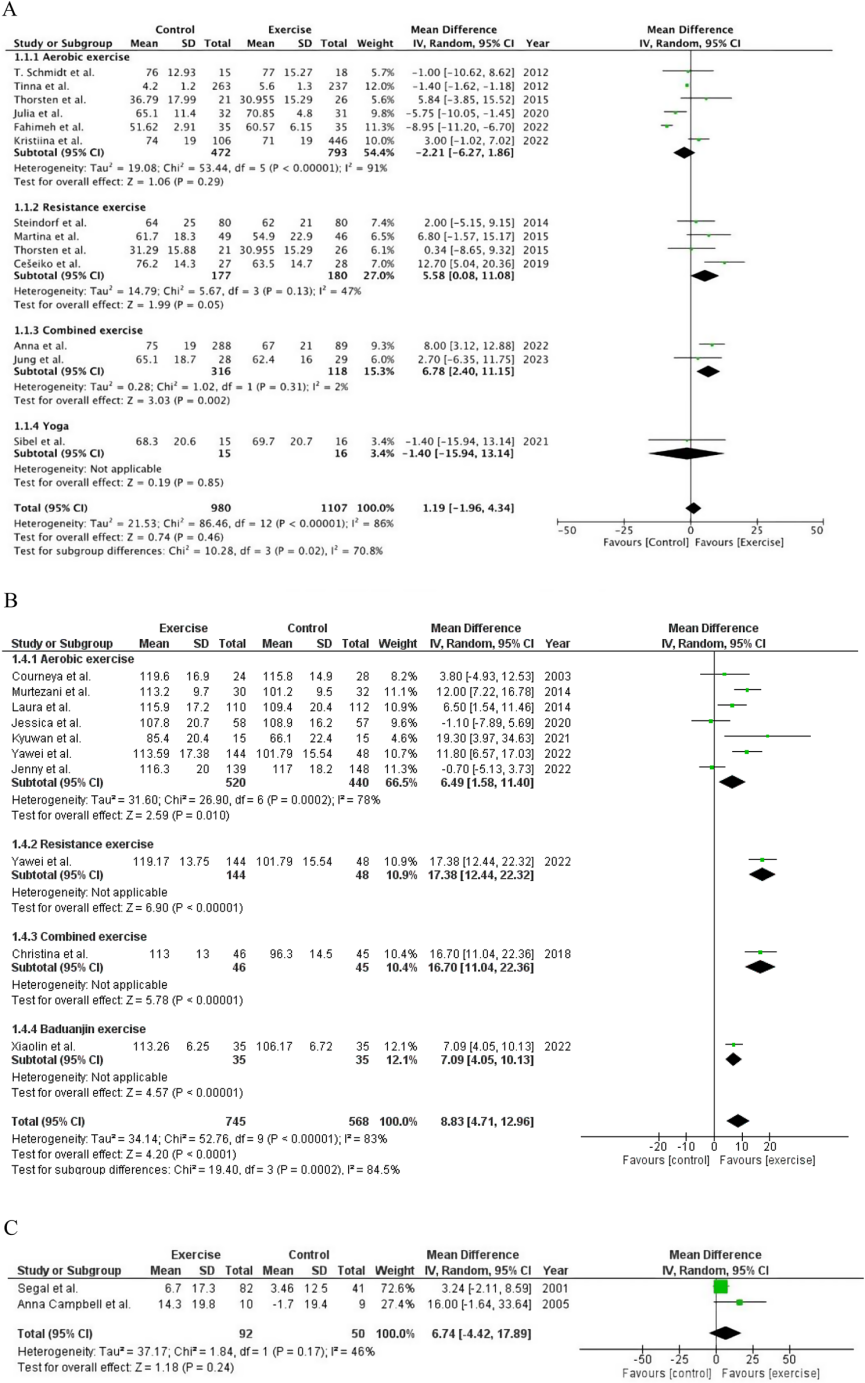
**A** Pooled results of HRQOL by EORTC QLQ-C30. **B** Pooled results of HRQOL by FACT-B scale (Direct post-intervention data). **C** pooled results of HRQOL by FACT-B scale (Mean change from baseline data)

**Fig. 5:**
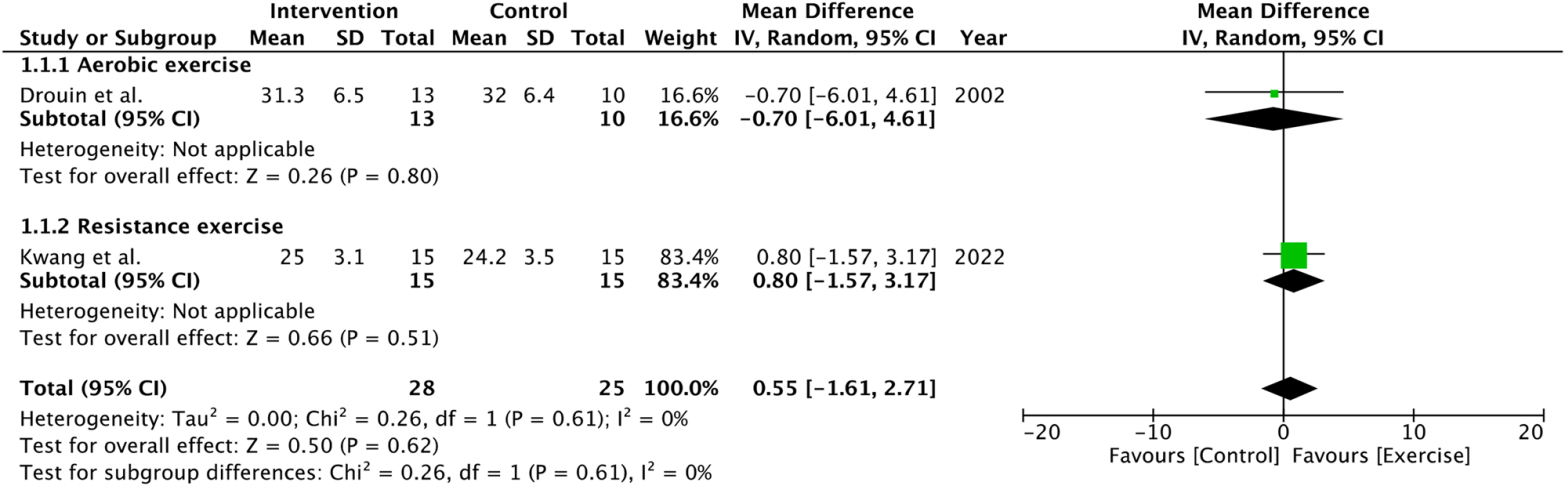
Pooled results from muscle strength by hand grip strength

**Fig. 6:**
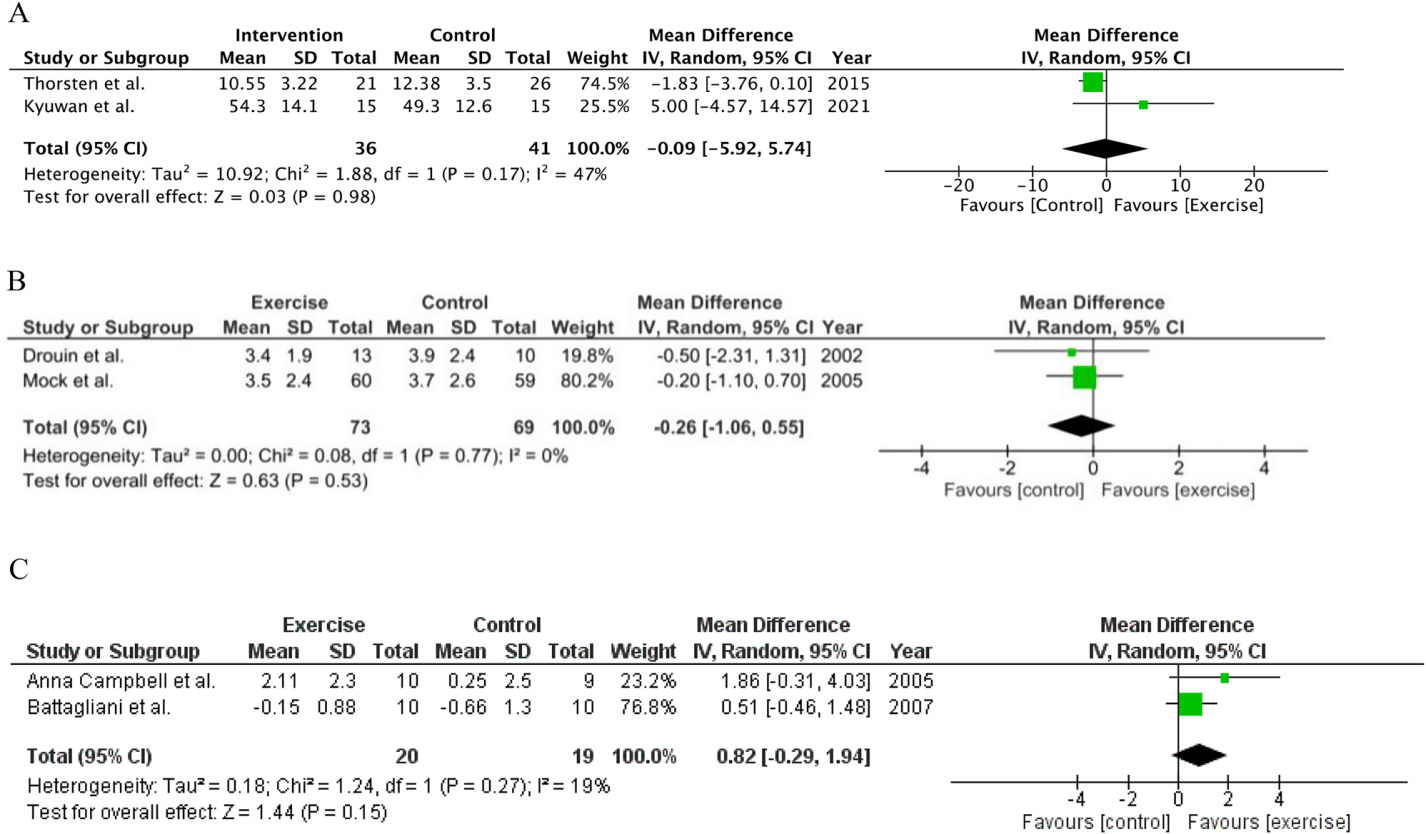
**A** Pooled results from Fatigue by MFI-20. **B** Pooled results from Fatigue by Revised Piper Fatigue scale (Direct post-intervention data). **C** Pooled results, Fatigue by Revised Piper Fatigue scale (Mean change from baseline data)

**Fig. 7:**
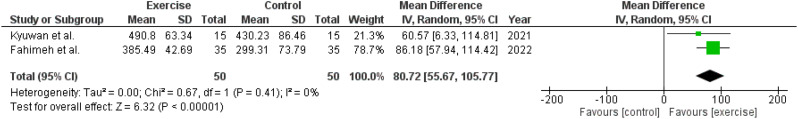
Pooled results from physical function by 6 MWT

Figures 3, 4, 5, 6 and 7 display the effects of exercise on health-related outcomes in patients with BC when compared with control (usual/standard care).

#### CRF

Eight studies evaluated the effect of exercise on CRF. Exercise did not significantly improved the CRF assessed by treadmill ergometer (*n* = 4) when compared with patients with BC receiving usual care (MD 4.96; 95% Cl [-2.79, 12.70]; *P* = 0.21; I^2^ = 96%). However, exercise significantly improved the CRF assessed by cycle ergometer (*n* = 4) compared with usual care (MD 2.07; 95% Cl [1.03–3.11]; *P* = 0.0001; I^2^ = 0%). Particularly, aerobic exercise group significantly improved the CRF when assessed by cycle ergometer compared with usual care (MD 2.48; 95% Cl [1.11, 3.84]; *P* = 0.0004; I ^2^ = 0%) Fig. 3A, B represents the analysis results of CRF.

#### HRQOL

HRQOL was monitored by twenty-five studies using two scales. Exercise did not significantly improve HRQOL assessed by EORTC QLQ C-30 scale (*n* = 13) compared with usual care. (MD 1.19; 95% Cl [-1.96, 4.34]; *P* = 0.46; I^2^ = 86%). However, combined exercise showed significant improvements in HRQOL assessed by EORTC QLQ-C30 scale compared with patients with BC receiving usual care (MD 6.78; 95% Cl [2.40, 11.15]; *P* = 0.002; I^2^ = 2%). In contrast, HRQOL assessed by FACT-B scale, using direct post-intervention values (*n* = 10), showed significant improvement in exercise group compared with usual care (MD 8.83; 95% Cl [4.71, 12.96], *P* < 0.0001; I^2^ = 83%). However, HRQOL assessed by FACT-B scale using mean change from baseline data (*n* = 2) showed non-significant improvements in exercise group (MD 6.74; 95% Cl [-4.42, 17.89]; *P* = 0.24; I^2^ = 46%). (Fig. 4A, B, C)

#### Muscle strength

The effect of exercise on muscle strength assessed by hand grip strength was demonstrated by two studies. Both studies showed non-significant improvements in exercise group compared with patients with BC undergoing usual care. (MD 0.55; 95% Cl [-1.61, 2.71]; *P* = 0.62; I^2^ = 0%). (Fig. 5)

#### Fatigue

Fatigue was monitored by six studies using two scales. MFI-20 scale (*n* = 2) demonstrated non-significant improvements in fatigue in exercise group compared with usual care group (MD -0.09; 95% Cl [-5.92, 5.74], *P* = 0.98; I^2^ = 47%). Fatigue assessed by Revised Piper Fatigue Scale using direct post-intervention (*n* = 2) and mean change from baseline data (*n* = 2) displayed non-significant improvements in the exercise group (MD -0.26; 95% Cl [-1.06, 0.55]; *P* = 0.53; I^2^ = 0% in direct post-intervention data and MD 0.82; 95%Cl [-0.29, 1.94]; *P* = 0.15; I^2^ = 19% in mean change from baseline data). (Fig. 6A, B, C)

#### Physical function

Physical function (*n* = 2) assessed by 6 MWT was significantly improved in the exercise group compared with the usual care group (MD 80.72; 95% Cl [55.67, 105.77]; *P* < 0.00001; I^2^ = 0%). (Fig. 7)

## Discussion

Our updated meta-analysis including over 3000 patients with BC outlines several key findings. First, exercise in patients with BC leads to significantly improved CRF. Second, exercise improved the HRQOL in patients with BC during and after treatment. Third, physical functioning was greatly improved in patients undergoing exercise. Lastly, the results of our meta-analysis indicate, combined exercise regimen including both aerobic and resistance training is more effective for improvement of health-related outcomes in patients with BC.

Our findings concur with prior meta-analyses evaluating the effects of exercise in patients with BC. However, the multiple short-comings of previous meta-analyses confer greater reliability to our results. Although prior meta-analyses and reviews have shown that exercise improves health-related outcomes including CRF, HRQOL and physical function in patients with BC [8, 9], they have often pooled different types of exercise like resistance training, strength training, yoga and aerobic exercise like treadmill and cycling were pooled together. Such analyses may lack accuracy and reliability owing to possible differences in outcomes with the type of exercise used. Similarly, patients with BC undergoing different treatments were pooled in the same analysis, this might cause changes in the results. In addition, some prior studies used different control groups [45] or had low statistical power owing to smaller number of participants or shorter durations of follow-up. Moreover, some prior studies evaluated HRQOL and fatigue using different questionnaires and scales which may have led to some reporting bias in their methodology [9].

Overall, our results showed that exercise improved CRF in patients with BC assessed with cycle ergometer. However, stratification by type of exercise yielded interesting findings whereby two different exercise techniques (treadmill versus cycle ergometer) yielded conflicting results for our exercise subgroups (aerobic, resistance or combined). Aerobic exercise training improved CRF by cycle ergometer while combined aerobic and resistance training did not yield any significant improvement. However, aerobic training yielded a non-significant result for CRF using the treadmill ergometer while combined aerobic and resistance training showed marked improvement in CRF. These contradictory findings using the treadmill versus cycle ergometer can possibly indicate some heterogeneity and methodological bias in assessing the maximal oxygen uptake ( VO_max_, key indicator of functional performance [46], since low VO_max_ indicates higher risk of mortality in advanced stages of BC [47] ) using the two techniques. While both cycle and treadmill ergometer are known tools for effective assessment of CRF, it should be noted that since they focus on assessing VO_max_, they are more likely a better choice for assessing aerobic training compared with resistance training. In addition, there is only one study for combined exercise training and no study for resistance training in specific to assess CRF. Hence, the results of combined training can possibly be ignored for this outcome. Nonetheless, the contradictory findings for CRF with aerobic training using cycle ergometer versus treadmill ergometer are important to consider and may suggest that aerobic training using cycle ergometer is more likely to benefit patients with BC undergoing treatment or post-treatment compared with treadmill ergometer.

HRQOL is an important parameter to be evaluated in patients with BC. Our results showed improvement in HRQOL overall. However, the results varied with the different types of scales used to assess HRQOL, namely FACT-B and EORTC QLQ-C30 scales. HRQOL assessment using FACT-B scale [48] demonstrated marked improvements across all exercise subgroups (aerobic, resistance and combined). Additionally, there was also evidence of HRQOL improvement with Baduanjin exercise, but more data is needed to confirm this since this result is based on one study only. However, these findings are based on direct post-intervention data from the included studies and does not account for mean change from baseline QOL status. Our pooled analysis of mean change from baseline data including only two studies showed a trend towards improvement in HRQOL but the results were not significant. Given that mean change from baseline is a more accurate way of assessing HRQOL, future studies need to assess HRQOL by taking the baseline status into account. Moreover, details of both baseline and post-intervention result should be provided in the trials. On the other hand, assessment of HRQOL by the EORTC QLQ-C30 scale [49] demonstrated little or no improvements with exercise in patients with BC. Stratification by exercise type suggested improved HRQOL with the combined exercise regimen only. Although exercise has shown improved HRQOL in patients with BC as suggested by our analysis and previous research [8], more studies with emphasis on strengths and weaknesses of assessment scales, and accountability of baseline QOL status should be considered for future research.

Exercise in patients with BC led to significant improvements in physical function assessed by 6-MWT. This is important since worsening of physical function reduces the risk of survival post-treatment [50]. In addition, evidence suggests that exercise provides protection against physical damage such as decreased muscle mass, muscle strength and joint mobility occurring in patients with BC post treatment [51]. However, our analysis presented non-significant improvements in muscle strength with exercise. Furthermore, cancer-related fatigue also affects physical function resulting from various factors including psychological, behavioral and biological, and is one of the most widely reported symptoms in patients with BC [52]. Fatigue assessed by MFI-20 and the revised piper fatigue scale showed little or no difference on patients with BC undergoing exercise regimen, although Thorsten el al [26]. and a meta-analysis in 2016 had shown significant improvement in fatigue in patients with BC undergoing exercise [9]. In future, research should be conducted to assess fatigue-related symptoms in patients with BC, since it can even prove to be fatal in patients with BC undergoing treatment or post-treatment.

Our findings summarize the available trial-level evidence and lay the basis for future research, with the pooled results raising multiple questions which need to be addressed so that clinicians can provide the best possible advice to patients with BC to help alleviate their symptoms. Although exercise can potentially improve HRQOL and physical function, it does not yield any significant benefits over fatigue-related symptoms, and the effects on CRF remain questionable. In future, RCTs should be conducted to evaluate the effects of exercise on CRF and fatigue-related symptoms in patients with BC undergoing treatment or post treatment. In addition, trialists should focus on exploring different types of exercises individually to evaluate which form of exercise is more beneficial for the patients. Although combined exercise seems to reap more beneficial effects than aerobic or resistance training alone, there is a dearth of evidence regarding its effectiveness in patients with BC, which should be evaluated further. Moreover, clinicians should scrupulously evaluate the short-comings of different exercise techniques and assessment scales, and uniformity must be ensured in the upcoming trials with respect to their methodological aspect of evaluating and monitoring the progress with exercise intervention.

The present meta-analysis has some limitations. First, direct post-intervention data was analysed because of lack of availability of data for mean change from baseline. This may have impaired our results as some patients may have had improved physical fitness at baseline. Second, various treatments were utilized along with some included studies assessed outcomes during treatment while others assessed outcomes after treatment which may have led to difference in results owing to treatment status. Third, not all exercise trials were supervised which can cause bias due to deviations from intended exercise. Fourth, long term effects such as adverse events were not evaluated which might limit any definitive conclusion. Fifth, the nature of self-reported questionnaires can lead to response bias. Sixth, significant heterogeneity was reported in analysis of HRQOL and CRF by treadmill ergometer particularly in aerobic exercise intervention which can be attributed to absence of studies, short duration of trials, low statistical power resulted from sample size and various methodologies of aerobic exercise regimen. Furthermore, our study used outcome measures for cancer-related fatigue with varying reliability due to data availability constraints. We relied on handheld dynamometry as a surrogate measure for whole-body muscle strength, and the 6-minute walk test to assess physical functionality as provided by the included studies, despite the availability of more specific assessment tools. These outcomes assessment might be considered unreliable and poorly supported by available psychometric data which can impair our findings. Finally, this is a study-level meta-analysis since individual patient data were not available.

## Conclusion

Exercise significantly improves the HRQOL, CRF and physical function in patients with BC during and after treatment. While the effects on HRQOL are consistent with all types of exercise namely aerobic, resistance or combined, the effects on CRF assessed by cycle and treadmill ergometer remain contradictory. Unfortunately, exercise failed to improve any fatigue-related symptoms and muscle strength. Although combined exercise training seems more likely to benefit patients with BC, there is limited evidence to make any confirmatory remarks in this regard. Well-powered RCTs with longer follow-up durations are needed to confirm our findings and resolve the conflicting findings related to exercise and health-related outcomes in this high-risk population.

### Electronic supplementary material

Below is the link to the electronic supplementary material.

Supplementary Material 1

## Data Availability

All data generated or analysed during this study are included in this published article and its supplementary information files.

## Abbreviations

BC: Breast cancer
RCT: Randomized control trials
QOL: Quality of life
CRF: Cardiorespiratory fitness
HTQOL: Health related quality of life
MD: Mean difference
CI: Confidence interval
SD: Standard deviation
ACSM: American College of Sports Medicine
DOX: Doxorubicin
