# Supplementary material for: Effectiveness of exercise modalities on breast cancer patient outcomes: a systematic review and meta-analysis
Source: Cardiooncology. 2024 Jun 18;10:38. doi: 10.1186/s40959-024-00235-z (PMC11184867; doi:10.1186/s40959-024-00235-z)

Supplementary File:

Supplementary Table 1. Summary of search strategy

Database	Search strategy	Results
MEDLINE	Search terms: (Exercise OR physical activity) AND (breast cancer patients OR breast cancer survivors)	5,086~~4876~~
Scopus	Search terms: (Exercise OR physical activity) AND (breast cancer patients OR breast cancer survivors)	4867
Google Scholar	Search terms: (physical training OR aerobic exercise OR resistance exercise OR physical activity OR combined exercise OR physical fitness OR supervised physical training) AND (breast cancer patients OR breast cancer survivors OR breast cancer neoplasm)	20,100
Cochrane	Search terms: (Exercise OR physical activity) AND (breast cancer patients OR breast cancer survivors)	3,217~~095~~
Web of Science	Search terms: (Exercise OR physical activity) AND (breast cancer patients OR breast cancer survivors)	1,579

Figure.1A Funnel plot assessing studies in analysis of HRQOL by EORTC QLQ-C30 scale.

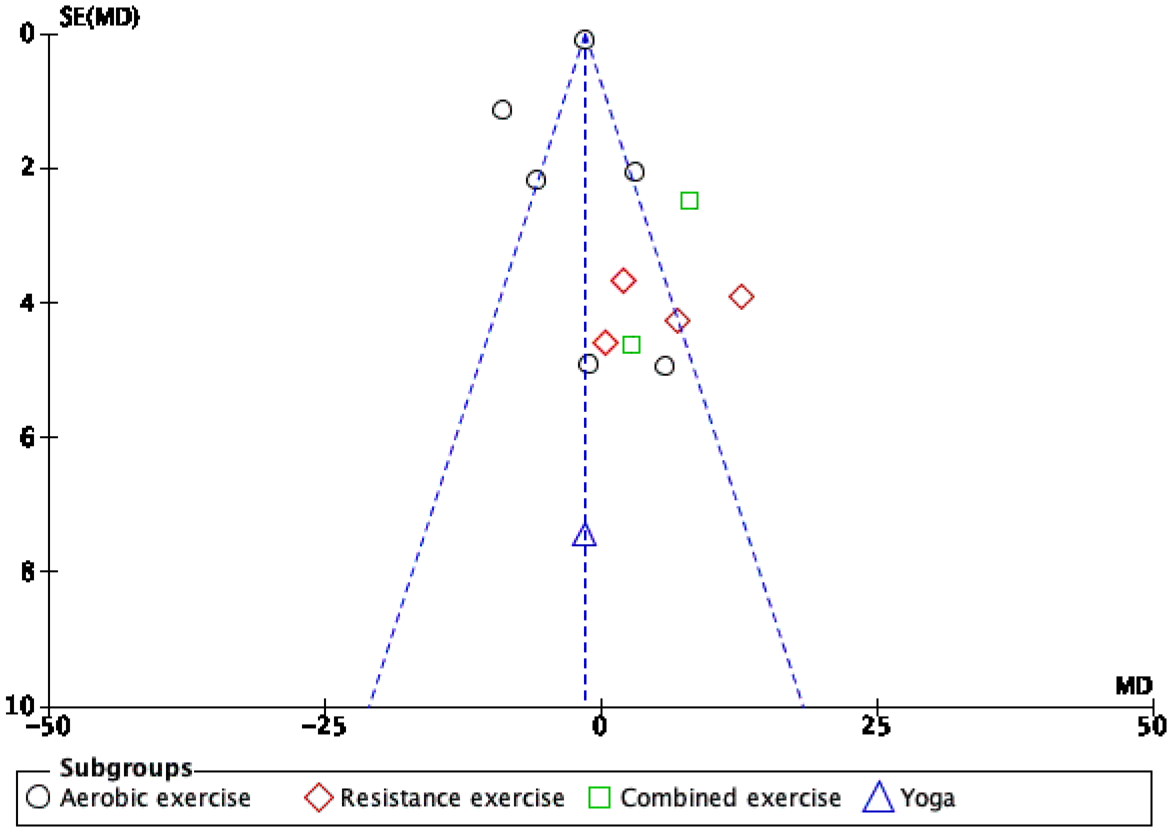

Figure.1B Funnel plot assessing studies in analysis of HRQOL by FACT-B scale.

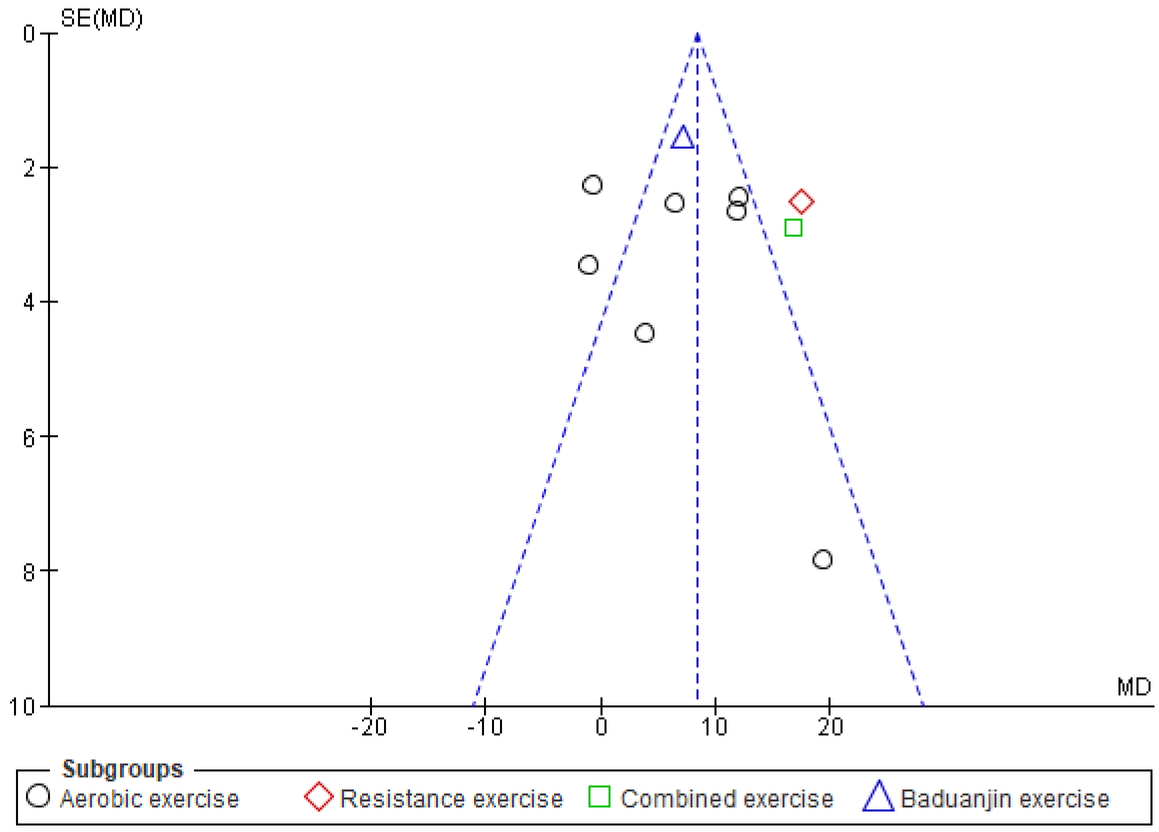

Supplement: Supplementary file 1 — Supplementary Material 1 [file 40959_2024_235_MOESM1_ESM.pdf]
